# Which pathways to psychiatric care lead to earlier treatment and a shorter duration of first-episode psychosis?

**DOI:** 10.1186/1471-244X-14-72

**Published:** 2014-03-12

**Authors:** Kamaldeep Bhui, Simone Ullrich, Jeremy W Coid

**Affiliations:** 1 Centre for Psychiatry, Wolfson Institute of Preventive Medicine, Barts & The London School of Medicine & Dentistry, Queen Mary University of London, London EC1M 6BQ, UK

**Keywords:** Psychosis, Early treatment, Anti-psychotics, Pathways, Schizophrenia & DSMIV diagnoses

## Abstract

**Background:**

The pathways to care in a first onset psychosis are diverse and may influence the chances of early treatment and therefore the duration of untreated psychosis. We test which pathways to care are associated with a delay in receiving treament and a longer duration of untreated psychosis (DUP).

**Methods:**

In a population based survey, we interviewed 480 people with first episode psychosis aged 18 to 64 years over a 2-year period. Information from structured interview and case files provided DSM-IV diagnostic, clinical, and demographic information. Consecutive contacts in the care pathway were mapped using the World Health Organisation’s Encounter Form. Using information from all sources, DUP was defined as time from symptom onset to first treatment with antipsychotic medication.

**Results:**

The most common first contacts were primary care physicians (35.2%), emergency rooms in general hospital settings (21.3%), and criminal justice agencies (25.4%). In multivariate regression models, compared to DUP for those first in contact with primary care, DUP was shortest for first encounters with psychiatric emergency clinics (RR = 0.4, 95% CI: 0.23-0.71) and longest for first encounters with criminal justice agencies (RR = 1.61, 95% CI: 1–2.58). Older age was associated with a longer DUP (RR = 1.01 per year, 95% CI: 1–1.04). A shorter DUP was associated with a diagnosis of mania and affective psychoses-NOS compared with schizophrenia (RR = 0.22, 95% CI: 0.14-0.35; RR = 0.18, 95% CI: 0.06-0.54, respectively), for Black compared with White ethnicity (RR = 0.52, 95% CI: 0.34-0.82), and for each close person in the social network (RR = 0.9, 95% CI: 0.84-0.96).

**Conclusions:**

To further reduce DUP, better links are needed between primary care, emergency rooms, criminal justice and psychiatric services.

## Background

Psychosis is associated with high levels of co-morbid psychopathology and premature mortality [1,2]. Patients presenting with psychotic disorders are usually young at first episode, and some may avoid prompt treatment if they lack insight; they can also experience poorer quality of life, impaired social functioning, more severe symptoms, a prolonged duration of untreated psychosis (DUP) and consequently a poorer long term prognosis with more relapses [3,4]. Therefore, reducing DUP has become the primary aim of modern psychiatric services for patients with a first episode of psychosis [4,5]. The purpose of early treatment is to reduce the period of time in which a patient’s life is disrupted and return them to full functioning as soon as possible. Consequently, most middle and high-income countries have introduced community-based services to improve accessibility of treatment alongside better links with non-health related community agencies [6,7].

In most high income countries a process of de-institutionalization has taken place alongside the development of community services [8]. The provision of emergency and crisis care has changed from dedicated psychiatric “emergency clinics” [9] staffed by mental health professionals and “emergency rooms” in general hospital settings towards a network of other community based agencies [10]. These include home treatment, crisis and early intervention teams [11-13] for which there is evidence of improved patient care, the prevention of admissions to hospital with marginal improvement in satisfaction with services [14].

In non-psychiatric medical specialities integrated care pathways are organised in order to standardise patient care and improve outcomes [15]. In psychiatric research, naturalistic care pathways are often described to show how patients seek help. A widely accepted model of naturalistic pathways to psychiatric care proposed that general practitioners were the first points of contact. General practitioners then refer to specialist psychiatric services if necessary [16]. However, general practitioners may be less commonly sought in the pathways for patients with a first episode of psychosis [17] and other agencies may also be sought [17-19]. Contact with some of these agencies, for example, the criminal justice system, are associated with a longer DUP [20].

This paper reports on naturalistic care pathways and DUP from the East London First Episode Psychosis Study (ELFEPS) and addresses the following research questions: (i) Which services/agencies are encountered by these patients in their pathways to specialist psychiatric care, (ii) which of these services/agencies and individual characteristics of these patients are independently associated with the shortest DUP.

## Methods

### Sample

The East London First Episode Psychosis Study (ELFEPS) is a large, population-based incidence study in three neighbouring local government boroughs in East London, UK. The three borough were City and Hackney, Newham, and Tower Hamlets. The study area was exclusively inner-city urban, characterized by high levels of socioeconomic deprivation. Historically, it has hosted a number of diverse migrant groups who settled in these borough over many years when coming to the United Kingdom. The study sample, data collection, design, consent and confirmation of ethical approval from the East London ethics committee have previously been reported in detail [21]. The study took place between December 1, 1996 and November 30, 1998 in City and Hackney; and from December 1, 1998, to November 30, 2000, in Newham and Tower Hamlets. This paper presents a re-analysis of data - originally collected for an incidence study- in order to investigate pathways to care and duration of untreated psychosis before the introduction of early intervention teams and when psychiatric emergency clinics in hospital settings were common.

We identified and screened everyone aged 18 to 64 years living in the study area if they made contact with psychiatric services for the first time (including adult community mental health teams, inpatient units, forensic services, learning disability services, adolescent mental health services, and drug and alcohol units). Health service bases were contacted weekly to identify all potential candidates. To minimise leakage tested methods [22] were applied during the study period to identify patients missed by the screening process, including checking with psychiatrists in private practice, private psychiatric hospitals served by the study area, and high-security hospitals, reviewing new service registration forms in the medical records department, and examining computerised information systems. The initial inclusion criteria were based on those used in the World Health Organization study and the Ætiology and Ethnicity in Schizophrenia and Other Psychoses study, except the AESOP study age range was 16–64 whereas our study was for 18–64 [23,24]. Patients in their first stages of illness meeting the inclusion criteria were identified and went on to subsequent stages of the protocol.

All patients given a diagnosis of any psychotic syndrome were identified and the cases were reviewed. Clinical professionals were contacted when there was uncertainty regarding cases. Patients who passed the screen underwent a battery of assessments including the Schedules for Clinical Assessment in Neuropsychiatry (SCAN) [25] the Personal and Psychiatric History Schedule (PPHS) [24]. This is a structured clinical interview used in the WHO studies; the data are checked across case records, hospital records and by interview where the patient rates the answers which are pre-coded [26]. A structured schedule gathered individual socio-demographic data. The duration of untreated psychosis (DUP) was estimated as the time from the first report of a psychotic symptom or any early symptom associated with the psychotic disorder to the time of first taking prescribed anti-psychotic medication. This information was taken from the PPHS. Any inconsistency of more than a month was resolved by consensus decision based on the research and clinical team considering all sources of information.

For all patients who declined interview the SCAN Item Group Checklist was completed on case notes and information from clinical staff. Researchers were trained in the SCAN interview on a World Health Organization–approved course to establish pre-study reliability using independent ratings of videotaped interviews. Diagnoses were allocated by consensus agreement between the principal investigator (J.W.C.) and clinical researchers who conducted the individual assessments. The researcher presented the clinical information to the principal investigator who remained blind to the individual characteristics of the patient. Diagnoses were made using this and information from the case notes, item ratings in the SCAN and collateral histories, all according to the DSM-IV [27].

Pathways to care were measured using the World Health Organization encounter form which documents the carer from whom help is initially sought (the first pathway contact), and then the next carer (second pathway contact), and finally a third pathway contact. This measure of pathways to care has previously been used in international studies of common mental disorders and psychotic disorders but few studies are large enough to present data by a range of specific services found in urban areas [6,28]. In this study, the types of pathway contacts were classified into the following groups: specialist psychiatric care (PS), psychiatric emergency clinics (EC) staffed by psychiatric specialists, emergency rooms (called accident and emergency departments in the UK; A&E) within general hospital settings, general practitioners in Primary Care (GP), community based health and social care by social workers and health visitors (CHSC), hospital medical and surgical services (HospMed), criminal justice agencies such as the police, prisons or solicitors (CJS); and native, lay and religious healers (NRH).

## Statistical analyses

All analyses were undertaken in STATA 11.0 [29]. Absolute (n) and relative frequencies (%) of people encountering a specific pathway contact were described; the proportions of people from any first pathway contact proceeding to services that were the second or third pathway contact were calculated and charted.

As individual characteristics may influence help seeking behaviour, pathways to care and DUP, the individual characteristics were first tabulated by the first contacts on the pathway to care. Chi-squared and exacts tests were used to identify which characteristics were statistically significantly associated with the pathways to care.

The individual characteristics were tabulated by DUP. The DUP (months) was summarised as a median and inter-quartile range in the total sample, and by specific characteristics: age groups, gender, single status (yes/no), qualifications (yes/no), social class (based on occupational group as social class I, II and III; IV and V; and unclassified if not employed), the number of close persons known (as a measure of support and social network), place of birth (UK/not UK), whether the patient was detained within a week of contact with services (yes/no), and by the first pathway contact. Detention within a week of contact was entered a covariate as this included compulsory treatment with anti-psychotic medication under the powers of mental health legislation (Mental Health Act in the UK). Therefore, those who are detained within a week of contact with services may have shorter DUP because of the behaviours leading to the decision to detain and treat against their will. Ethnicity was ascribed on the basis of a multi-ethnic panel of researchers using all available information including self acription, place of birth, and parental place of birth, the final decision being that of the researcher. The ethnic codes were those of the 2001 census categories grouped for analysis to Black, White, Indian subcontinent and Other, and, where necessary, into smaller groups to investigate sub-group effects (White British, White Other, Black Caribbean, Black African, Indian, Pakistani, Bangladeshi, Other). Kruskal-Wallis non-parametric statistical tests were used to compare univariate distributions of DUP by individual demographic and clinical characteristics, and then by the pathways to care (first contacts as these were critical rather than second or third contacts).

All data were double entered and classifications and entries were checked by two independent researchers before the study dataset was ‘locked’. As the data we used were largely descriptive, the validity was checked by cross-referencing the hospital records with patient reports during the research interview. Poisson regression models were applied to provide univariate and fully adjusted multivariate estimates of associations [Rate Ratio (RR), 95% confidence intervals (95% CI)] between DUP (months) and individuals’ demographic and clinical characteristics and the pathways to care. Poisson regression was selected for a number of reasons. It can be used for count, binary and as an alternative to Cox’s proportional hazhards for ‘time to event’ data [30]. Other advantages of poisson regression include that it provides Risk Ratios which are preferred as odds ratios can be misleading [31]. DUP is the outcome measured in the number of months before anti-psychotic treatment, this effectively amounts to a single count of number of months. The ‘vce (robust)’ command was specified for the Poisson regression models in STATA. This provides robust estimates of standard errors and the approach is recommended over log-linear analyses and with over-dispersion, zero inflated data, and even if not all the assumptions of a Poisson distribution are met [32,33].

## Results

### Descriptive epidemiology of pathways to psychiatric care

The total cohort of 480 people with a first-episode psychosis had complete data on the pathway to specialist psychiatric care. Cumulatively, of the total cohort of 480 people, 15 (3.13%), 352 (73.33%) and 469 (97.71%) were in contact with psychiatric services at the first, second and third pathway contacts, respectively. Consecutive psychiatric contacts were not counted as these were likely to reflect internal referrals between services. DUP was not related to number of pathway contacts (Kruskal Wallis X^2^ = 0.78, df = 2, p = 0.49) and so we concentrated on the first pathways contact as the greatest potential source of variation in DUP.

**Figure 1:**
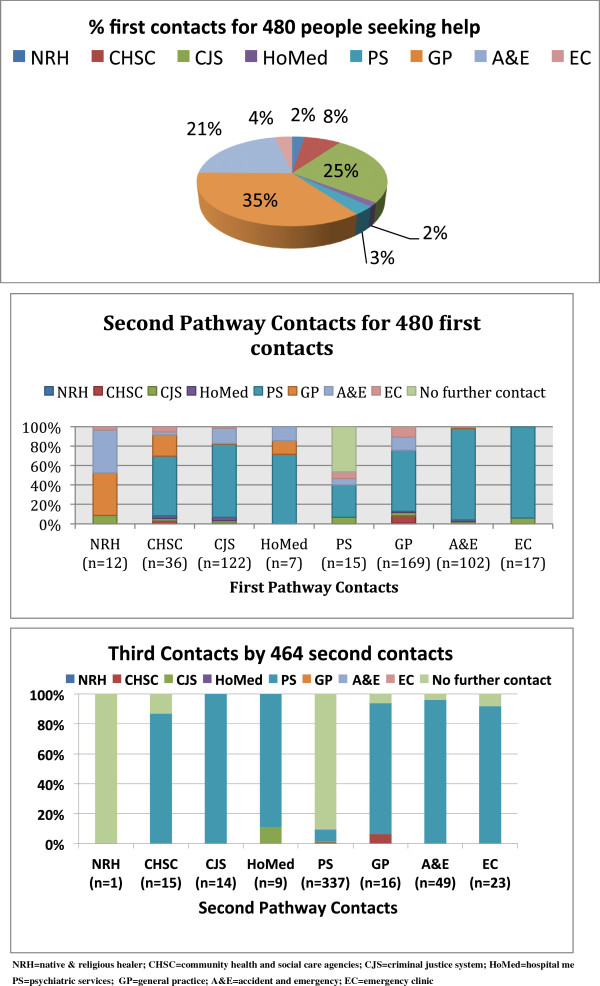
Pathways to psychiatric care.

The most common first contacts were GPs, A&E, and CJS agencies (see Figure 1). Fifteen people encountered psychiatric services as a first pathway contact. Of those with non-psychiatric first contacts (n = 465), only one person had no second contact. Of these 464 patients proceeded to a second pathway contact (see Figure 1); of these 337 (72.46%) encountered specialist psychiatric services as the second pathway contact. This left a further 127 people who had not yet contacted psychiatric services at first or second contact. Of these, 8 did not have a third pathway contact. Therefore, 119 patients proceeded to a third contact of whom 117 then encountered psychiatric services, one encountered CJS agencies, and one encountered CHSC agencies. At the time of the study all were in contact with services, so it is likely that those not having a second or third contact (total of 9 patients) did ultimately encounter psychiatric services but reported they did not seek further help.

Table 1 shows individual characteristics of patients according to their first contacts on the pathway to psychiatric care. For this cross-tabulation the pathways contacts were recoded to binary variables (e.g. native and religions healers versus the rest; primary care versus the rest, etc.) and the diagnosis was recoded to ‘schizophrenia vs all other diagnoses’.

**Table 1:** Each service (versus all others) on the care pathway: demographic and clinical characteristics

**Contacts on care pathway**			**GP**	**NRH**	**CHSC**	**CJS**	**HospMed**	**PS**	**A&E**	**EC**
**Characteristics**		**N**	**n (row%) and P values (X**^**2 **^**or Exact test)**							
Age	25 or less	166	58 (34.94)	4 (2.41)	9 (5.42)	44 (26.5)	1 (0.6)	7 (4.22)	39 (23.49)	4 (2.41)
	>25 to 32	159	49 (30.82)	4 (2.52)	14 (8.81)	49 (25.16)	2 (1.26)	1 (0.63)	44 (27.67)	5 (3.14)
	>32	155	62 (40.00)	4 (2.58)	13 (8.39)	38 (24.52)	4 (2.58)	7 (4.52)	19 (12.26)	8 (5.16)
			*P = 0.23*	P = 1.00*	*P = 0.45*	*P = 0.92*	P = 0.27*	P = 0.06*	*P = 0.003*	P = 0.43*
Gender	Male	294	81 (27.55)	8 (2.72)	21 (7.14)	97 (32.99)	2 (0.68)	10 (3.40)	63 (21.43)	12 (4.08)
	Female	186	88 (47.31)	4 (2.15)	15 (8.06)	25 (13.44)	5 (2.69)	5 (2.69)	39 (20.97)	5 (2.69)
			*P < 0.001*	P = 0.77*	*P = 0.71*	*P < 0.001*	P = 0.12*	P = 0.79*	*P = 0.90*	P = 0.46*
Single	No	202	91 (45.05)	7 (2.52)	21 (10.40)	31 (15.35)	4 (1.98)	4 (1.98)	42 (20.79)	4 (1.98)
	Yes	278	78 (28.06)	5 (2.48)	15 (5.40)	91 (32.73)	3 (1.08)	11 (3.96)	60 (21.58)	13 (4.68)
			*P < 0.001*	P = 1.00*	*P = 0.04*	*P < 0.001*	P = 0.46*	P = 0.29*	*P = 0.83*	P = 0.14*
Qualifications	No	255	88 (34.51)	8 (3.14)	22 (8.63)	69 (27.06)	5 (1.96)	4 (1.57)	51 (20.00)	8 (3.14)
	Yes	214	75 (35.05)	4 (1.87)	14 (6.54)	52 (24.30)	1 (0.47)	11 (5.14)	48 (22.43)	9 (4.21)
			*P = 0.90*	P = 0.56*	*P = 0.40*	*P = 0.50*	P = 0.23*	P = 0.04*	*P = 0.52*	P = 0.62*
Social class	I,II,III	113	46 (40.71)	1 (0.88)	6 (5.31)	29 (25.66)	1 (0.88)	2 (1.77)	23 (20.35)	5 (4.42)
	IV,V	226	73 (32.30)	6 (2.65)	15 (6.64)	61 (26.99)	3 (1.33)	6 (2.65)	53 (23.45)	9 (3.98)
	Unclassified	141	50 (35.46)	5 (3.55)	15 (10.64)	32 (22.70)	3 (2.13)	7 (4.96)	26 (18.44)	3 (2.13)
			*P = 0.31*	P = 0.42*	*P = 0.22*	*P = 0.65*	P = 0.79*	P = 0.37*	*P = 0.50*	P = 0.56*
Born in UK	No	*239*	*86 (35.98)*	*9 (3.77)*	*16 (6.69)*	*52 (21.76)*	*5 (2.09)*	*8 (3.35)*	*57 (23.85)*	*6 (2.51)*
	Yes	*241*	*83 (34.44)*	*3 (1.24)*	*20 (8.30)*	*70 (29.05)*	*2 (0.83)*	*7 (2.9)*	*45 (18.67)*	*11 (4.56)*
			*P = 0.72*	P = 0.09*	*P = 0.51*	*P = 0.07*	P = 0.28*	*P = 0.78*	*P = 0.17*	P = 0.32*
Ethnicity	White	177	*65 (36.72)*	*3 (1.69)*	*11 (6.21)*	*37 (20.90)*	*4 (2.26)*	*5 (2.82)*	*40 (22.60)*	*12 (6.78)*
	Black	160	*43 (26.88)*	*0*	*13 (8.13)*	*60 (37.50)*	*2 (1.25)*	*5 (3.13)*	*33 (20.63)*	*4 (2.5)*
	Indian sub-continent	114	*54 (47.37)*	*8 (7.02)*	*7 (6.14)*	*13 (11.40)*	*1 (0.88)*	*5 (4.39)*	*25 (21.93)*	1 (0.88)
	Other	29	17 (24.14)	1 (3.45)	5 (17.24)	12 (41.38)	0	0	4 (13.79)	0
			*P = 0.003*	P = 0.002*	P = 0.21*	*P < 0.001*	P = 0.81*	P = 0.80*	P = 0.78*	P = 0.04*
Diagnosis of schizophrenia	Yes	165	45 (27.27)	5 (3.03)	16 (9.70)	53 (32.12)	1 (0.61)	6 (3.64)	36 (21.82)	3 (1.82)
	No	315	124 (39.37)	7 (2.22)	20 (6.35)	69 (21.90)	6 (1.90)	9 (2.86)	66 (20.95)	14 (4.44)
			*P = 0.008*	P = 0.56*	*P = 0.19*	*P = 0.02*	P = 0.43*	*P = 0.64*	*P = 0.83*	P = 0.19*
Detained within a week	Yes	253	78 (30.83)	7 (2.77)	22 (8.70)	78 (30.83)	6 (2.37)	9 (3.56)	45 (17.79)	8 (3.16)
	No	221	89 (40.27)	5 (2.26)	13 (5.88)	42 (19.00)	1 (0.45)	6 (2.71)	56 (25.34)	9 (4.07)
			*P = 0.03*	P = 0.78*	*P = 0.24*	*P = 0.003*	P = 0.13*	*P = 0.60*	*P = 0.05*	P = 0.63*
Close perons^	mean (sd)	433	4.3 (4.15)	5.64 (4.70)	2.20 (2.32)	3.83 (3.63)	3.83 (2.99)	2.71 (2.76)	4.91 (4.80)	2.77 (1.99)

Note. *Exact test.

^Kruskal Wallis X^2^ = 19.51, df = 7 *P = 0.007.*

Those attending emergency rooms in general hospital setting at first contact were likely to be younger. Those first in contact with general practices were more likely to be women, did not have a diagnosis of schizophrenia, were not compulsorily detained within a week of contact with psychiatric services, and were less likely to be single or Black. Those in first contact with criminal justice agencies were more likely to be single, male, Black, and to received a diagnosis of schizophrenia, and to be detained within a week of psychiatric contact. When the specific Black groups were included instead of an overall Black category, both Black African and Black Caribbean groups had a greater risk of a first contact with criminal justice agencies compared with the White British group (Black Caribbean: RR = 2.11, 1.34 to 3.31, p = 0.001; Black African: RR = 1.65, 1.03 to 2.64, p = 0.04), with no significant differences for primary care contacts.

Data on the number of close persons was available for 433 people. The mean (standard deviation) number of close persons for each first contact are presented in Table 1. The more close persons in the social network, the less likely the patient was to first contact emergency clinics (RR = 0.64 per close person, 95% CI: 0.45-0.93, P = 0.02) and community health and social care agencies (RR = 0.51 per close person, 95% CI: 0.35-0.75, P = 0.001). No other associations between specific first pathway contacts and the number of close persons reached statistical significance.

### Duration of untreated psychoses

Table 2 shows trends in DUP in months according to demographic and clinical factors, and by specific first contacts on the pathway to psychiatric care. The minimum DUP of 1 month was reported by 30% of the cohort signifying treatment within a month of symptom onset. DUP was longer for men, patients with a diagnosis of schizophrenia, delusional disorder or depressive type of affective psychosis, and for certain first contacts on the care pathway: medical and surgical services in hospitals, criminal justice agencies, native and religious healers, and community based health and social care.

**Table 2:** DUP (months) and association with demographic and clinical characteristics

		**N**	**Median (inter-quartile range)**	**Kruskal Wallis Χ**^**2**^	**df**	**P**
Total sample		480	3 (1 – 9.5)			
Age	25 or less	166	3 (1 to 8)			
	>25 - 32	159	3 (1 to 10)	0.445	2	0.80
	>32	155	3 (1 to 10)			
Gender	Male	294	4 (1 to 10)	3.84	1	0.05
	Female	186	3 (1 to 8)			
Single	Yes	202	3 (1 to 9)	0.94	1	0.36
	No	278	3 (1 to 10)			
Qualifications	None	255	4 (1 to 10)			
	Yes	214	3 (1 to 8)	1.69	1	0.19
Social class	I, II, III	113	4 (1 to 9)			
	IV, V	226	4 (1 to 12)	6.99	2	0.03
	Unclassified, not working	141	3 (1 to 6)			
UK born	No	239	3 (1 to 9)	2.79	1	0.10
	Yes	241	4 (2 to 10)			
Ethnicity	White	177	4 (2 to 10)	5.08	3	<0.001
	Black	160	3 (1 to 5)			
	Indian sub-continent	114	4 (2 to 12)			
	Other	29	4 (2 to 12)			
Diagnosis	Schizophrenia	165	6 (2 to 12)	98.87	7	<0.001
	Delusional disorder	29	6 (4 to 18)			
	Brief psychotic disorder	46	1 (1 to 1)			
	Schizoaffective disorder	91	3 (1 to 9)			
	Non-affective psychosis NOS	29	2 (1 to 6)			
	Psychotic depression	67	5 (2 to 9)			
	Psychotic mania	49	1 (1 to 3)			
	Affective psychosis - NOS	3	1.5 (1 to 3)			
Detained within a	No	253	4 (1 to 12)	5.86	1	0.02
week of contact	Yes	221	3 (1 to 6)			
1st contact on	GP	169	4 (2 to 10)	13.02	7	0.07
care pathway	NRH	12	3 (1 to 9.5)			
	CHSC	36	5.5 (2 to 12)			
	CJS	122	3 (1 to 10)			
	HoMed	7	1 (1 to 3)			
	PS	15	3 (1 to 6)			
	A&E	102	2 (1 to 6)			
	EC	17	1 (1 to 6)			

Spearman correlation coefficient between the number of close persons and DUP = −0.28, P<0.001.

The shortest DUP was found for patients first presenting to general practices, psychiatric services, emergency rooms in general hospital settings, and emergency clinics staffed by psychiatric specialists. DUP correlated negatively with the number of close persons (Spearman’s correlation coefficient = −0.28, P<0.0001). Short DUPs were also reported among Black people (compared with White) and those detained within a week of contact with psychiatric services. When more specific Black ethnic groups were included, a shorter DUP was found only for the Black African group (RR = 0.52, 0.31 to 0.86, p = 0.01).

Of the total sample, 253 were compulsorily detained within a week of contact with services. This was included to capture acute and disturbed presentations that may lead to a shorter DUP because of emergency compulsory treatment. Data checks confirmed this. Detention commonly reflected violent behaviour or threatening behaviour at presentation; 98 of 253 people detained had been violent at presentation compared with 44 of 221 people not detained (38.74% vs 19.91% respectively; X^2^ = 19.92, df = 1, P < 0.001). 178 of 253 detained within a week had presented with threatening conduct compared with 104 of 221 not detained (70.36% vs 47.06% respectively, X^2^ = 26.68, df = 1, P < 0.001). Self-harm was not significantly associated with detention within a week of contact with services (X^2^ = 1.29, df = 1, P = 0.26).

Table 3 shows associations between DUP, demography, and clinical characteristics in a multivariate model that adjusts for potential confounding. Older age, being single, and having first contact with criminal justice agencies (compared with primary care) were each independently associated with longer DUP. Shorter DUP was found for first contact with emergency clinics (compared with primary care), those with diagnoses of either mania or affective psychosis not-otherwise specified (compared with schizophrenia), those with a greater number of close persons (per person), those detained within a week of contact (compared with those not detained within a week), and for Black compared with White people. When more specific ethnic groups were included in the model, only the Black African group had a statistically significantly lower DUP (RR = 0.51, 0.26 to 0.99, p = 0.05). The point estimate for the Black Caribbean suggested a shorter DUP compared with the White group, but the finding did not reach statistical significance (RR = 0.70, 0.41 to 1.19).

**Table 3:** Poisson regression with DUP as an outcome: univariate and multivariate analyses

		**Univariate**		**Multivariate**	
**Total sample**		**Rate ratio (95% CI)**	**P**	**Rate ratio (95% CI)**	**P**
Age	Per year	1.02 (1–1.03)	0.09	1.01 (1.00–1.04)	0.14
Gender	Male	1		1	
	Female	0.9 0.62–1.30)	0.58	1.07 (0.77–1.48)	0.69
Single	No	1		1	
	Yes	1.39 (0.99–1.97)	0.06	1.46 (0.99–2.14)	0.06
Qualifications	None	1		1	
	Yes	0.79 (0.56–1.13)	0.20	0.99 (0.72–1.37)	0.95
Social class	I,II,III	1		1	
	IV,V	1.15 (0.77–1.71)	0.51	1.07 (0.71–1.60)	0.75
	Unclassified, not working	1.16 (0.69–1.96)	0.57	1.31 (0.75–2.32)	0.34
Number of close persons	Per person	0.9 (0.84–0.96)	0.002	0.93 (0.89–0.99)	0.03
UK born	No	1		1	
	Yes	0.93 (0.65–1.32)	0.68	0.88 (0.60–1.29)	0.52
Ethnicity	White	1		1	
	Black	0.57 (0.38–0.84)	0.005	0.52 (0.34–0.82)	0.004
	Indian sub-continent	0.92 (0.58–1.45)	0.72	0.88 (0.54–1.44)	0.61
	Other	0.96 (0.47–1.96)	0.91	0.75 (0.39–1.47)	0.41
Schizophrenia		1		1	
Delusional disorder		1.64 (0.86–3.14)	0.13	1.59 (0.90–2.82)	0.11
Brief psychotic disorder		0.66 (0.27–1.64)	0.37	0.74 (0.31–1.78)	0.50
Schizoaffective disorder		0.6 (0.38–0.95)	0.03	0.75 (0.44–1.26)	0.28
Non-affective psychosis: NOS		0.67 (0.35–1.28)	0.22	0.6 (0.26–1.38)	0.23
Psychotic depression		0.84 (0.49–1.43)	0.52	0.91 (0.53–1.55)	0.72
Psychotic mania		0.19 (0.13–0.27)	<0.001	0.22 (0.14–0.35)	<0.001
Affective psychosis-NOS		0.16 (0.09–0.31)	<0.001	0.18 (0.06–0.54)	0.002
Detained within a	No	1		1	
week of contact	Yes	0.68 (0.47–0.98)	0.04	0.66 (0.45–0.96)	0.03
1st Contact on Pathway	Community health & social care	1.08 (0.65–1.79)	0.77	0.96 (0.54–1.69)	0.88
	Criminal justice system	1.28 (0.81–2.00)	0.30	1.61 (1.00–2.58)	0.05
	Hospital medicine	2.44 (0.44–13.42)	0.30	2.32 (0.88–6.16)	0.09
	Psychiatric services	0.85 (0.27–2.65)	0.78	0.81 (0.29–2.27)	0.68
	Accident & Emergency	0.84 (0.52–1.33)	0.45	0.84 (0.52–1.34)	0.46
	Emergency clinics	0.42 (0.24–0.72)	0.002	0.40 (0.23–0.71)	0.002

Note. R^2^ = 22.3% of variance on 427 observations. Data point for detained within a week of contact complete for only 474. Data point for close persons has only 433 observations. All Poisson estimates used Poisson regression commands in STATA with the vce (robust) command to ensure robust confidence intervals and guarding against over-dispersion and violations of model assumption.

## Discussion

### Care pathways and DUP

Reducing DUP has become the primary aim of modern psychiatric services for patients with a first episode of psychosis [4,5]. Consequently, most middle and high income countries have introduced community based services to improve accessibility of treatment with better links with community based non-health agencies [6,7]. This shift in resources has meant a reduction in hospital beds and emergency clinics, and less reliance on accident and emergency departments as community teams such as early intervention and crisis services are intended to provide better alternatives. The implication is that community teams are closer to the patients in need, more accessbile and therefore will be associated with a shorter DUP. In addition, data for this study were collected prior to the introduction of these new teams and so are instructive as we can compare current trends in DUP following the introduction of these teams with past DUP. Contrary to expectation we found that first contacts with emergency clinics staffed by specialist psychiatric teams resulted in shortest DUP, with longer DUP for pathways involving community agencies. In inner city urban areas that are densely populated but with a high prevalence of socially isolated individuals, 24 hour emergency clinics may be the most effective and rapid way of providing intervention [34]. However, psychiatric emergency clinics have been progressively phased out in the UK with funding shifted to community-based services, a policy trend that may need review in inner-city urban areas [35].

The next shortest DUP was found for first contacts with general practices, psychiatric services, and emergency rooms in general hospitals. If commissioners and providers of services aim to integrate care pathways to reduce DUP, links are needed between these services in order to minimize contact with services associated with longer DUP. A previous study of first onset patients estimated that a third had previous contact with criminal justice agencies making this an important modifiable component of the pathway into care [36]. Those working in these settings should be encouraged to initiate prompt referral to emergency rooms in general hospitals or general practice and then to psychiatric services. Although diversion schemes from the criminal justice system to hospitals have been introduced, these usually rely on the availability of inpatient facilities; where general hospital or psychiatric hospital beds are needed; their shortage will work against early diversion.

European and World Health Organization models of pathways to psychiatric care [7,37] are well-established but rarely investigated the effectiveness of different pathways for people with first episodes of psychosis. Our study shows that even when emergency clinics existed, the criminal justice system (police, lawyers and prisons), emergency rooms in general hospitals, and general practitioners were more likely to be contacted [19]. Consultations with native and religious healers are reported to be more common amongst immigrants from South Asia and Africa [6], but this was uncommon in this ethnically diverse inner London sample. Our findings suggest that this pathway was associated with a longer DUP and our findings suggest it should be avoided. Although this might be explained by under-reporting of non-conventional pathways, previous studies using the WHO Encounter Form have successfully identified non-conventional pathways [6,28]. Practitioners and mental health services must make sustained efforts to to improve the accessibility of services, and knowledge about services in communities.

### Comparisons with other studies

There was wide variation in DUP in the overall sample with medians ranging from 1 to 12 months. The median indicates how rapidly half of the cohort received anti-psychotic medication. It is more helpful when the distribution of DUP is negatively skewed and is less sensitive to outliers than a mean. Despite pathway variations, and that the data were collected before the new early intervention services had been established, the median DUP was 3 months, comparing favourably with other studies. In two systematic reviews [17,19] 20-33% of studies found a median DUP of below 3 months, with the majority reporting more than 3 months. One study [38] also located London was linked to an international centre of research with commensurate resources for service delivery, reported a median DUP of just over two months. A more recent study of first episode psychosis presentations to early intervention services found DUPs that were comparable with our findings [39]. Consistent with findings of a previous study in London [38] we found a shorter DUP specifically in Black groups (especially Black Africans); and like a recent early intervention in psychosis study [39] we found more criminal justice system contact for Black patients. This association with criminal justice system agencies requires more research as this is potentially modifiable if offending is motivated by psychotic symptoms. Future studies will need to investigation the reasons for a criminal justice system pathway for black patients. For example, is this more due to more offending, more violent offending, or to discrimination in the court process, or that community agencies are less effective at managing some patient groups who end up in the criminal justice system [40,41].

### Individual characteristics and DUP

Further studies are needed that compare DUP across different diagnostic groups within the psychosis spectrum [42]. Some diagnoses (DSM-IV mania and affective psychosis-NOS) were associated with shorter DUP. As we adjusted for detention in hospital that is associated with violence and other acute presentations, these can not explain the shorter DUP in manic presentations that might present a more conspicuous illness. Most previous studies compare DUP among people with a diagnosis of schizophrenia with those in a combined category where schizoprhenia is aggregated with other psychoses. As this comparison group might include affective psychoses with a shorter DUP, these studies may overestimate the relative DUP in schizophrenia compared with other psychoses. So the estimates of DUP may be too pessimistic, if the comparison group includes affective psychoses. Delusional disorders appear to have as long a median DUP as schizophrenia (in univariate data) although this finding is not sustained in multivariate models.

Black people had shorter DUP than those classified as White. The study showed that Black patients do not inevitably face delays on pathways to anti-psychotic treatment, an assumption previously put forward to explain their higher rates of admission and detention [18,41]. Indeed, Black people were treated more effectively and earlier, challenging the notion that services are not accessible and ineffective. These finding held despite adjustment for diagnosis, and the number of close persons, which has previously been show to faciliate help-seeking [43] and avoid detention. A prolonged DUP was associated with a diagnosis of delusional disorder, and fewer close persons [20]. We also adjusted for detention within a week of contact with services, so acutely disturbed presentations cannot account for this finding either.

### Methodological considerations

This is one of the largest studies of pathways to care for a first episode psychosis cohort, and made use of structured research instruments rather than clinical service definitions of first episode psychosis. Many comparative studies are underpowered, describe pathways among small numbers of individuals in one or two ethnic groups, do not consider DUP for affective psychoses and do not test the relationship between specific pathways and DUP. Given the study involved a retrospective account of consecutive contacts, it is essentially a cross-sectional study in which the causal effects can not be determined with certainty. At the time of the study, all patients were already in contact with mental health services and on treatments so poor recall due to delusions and hallucinations is an unlikely source of information bias.

Statistical tests can not disentangle potential explantory or mediating influences from confounding influences. Although a prospective population study might better at discerning the causal direction, as incident psychotic disorders are rare such a study would require a very large sample size and study duration and cost. However, the mechanism through which specific pathways were chosen and factors that may influence help seeking like health beliefs require more in-depth qualitative studies.

## Conclusion

Patients attending emergency clinics had the shortest DUP and those attending criminal justice agencies had the longest DUP. The place of diversion to hospitals, emergency clinics and emergency care need review. Black patients had a shorter DUP so their contact with criminal justice agencies can not be fully explained by delays in seeking help and warrant further research.

## Competing interests

The authors declared that they have no competing interests.

## Authors’ contributions

KB had full access to all of the data in the study and takes responsibility for the integrity of the data and the accuracy of the data analysis. KB undertook analyses, developed the analysic strategy and prepared the manuscript, consecutive and final drafts. JWC (as PI) and SU were responsible for collecting the original data, and commenting on and editing consecutive drafts, and refining the analytic strategy. All authors read and approved the final manuscript.

## Pre-publication history

The pre-publication history for this paper can be accessed here:

http://www.biomedcentral.com/1471-244X/14/72/prepub
